# Lego® bricks assisted training of the novice debriefers

**DOI:** 10.12688/mep.19314.1

**Published:** 2023-03-20

**Authors:** Emanuele Capogna, Pier Luigi Ingrassia, Giorgio Capogna

**Affiliations:** 1 EESOA, Simulation Center, Roma, 00151, Italy; 2 CeSi, Simulation Center, Lugano, 6900, Switzerland

**Keywords:** simulation, debriefing, Lego Serious Play, healthcare simulation, high fidelity simulation, debriefers training, simulation scenario, strategic dialogue

## Abstract

Lego® Serious Play® is a guided workshop in which participants construct Lego creations to represent symbolic and metaphorical ideas in response to assignments. How to encourage inexperienced debriefers to concentrate on dialogue and communications strategies rather than engage in an unstructured debate on technical or behavioral abilities is one of the main challenges in training people to debrief a high-fidelity simulation session.

We explore the use of Lego bricks in this study to build straightforward, standardised situations that debriefers in training can use to practice leading discussion. With this method, the different debriefing methodologies may be practiced focusing exclusively on method and dialogue, without getting involved or having to concentrate on the technical aspects.

## Introduction

### The background: Lego® Serious Play®

In the mid-1990s, Lego experienced a decline in sales as a result of changes brought about by the success of new forms of play for children such as video games. To curb and solve the problem, it was suggested that changes in strategy should be introduced by implementing new tools and methodologies in management decision-making and by abandoning the old methods ^1– 3^ ( http://s-play.eu/en/news/).

Lego called in consultants, Johan Roos and Bart Victor, who came up with the concept and method of serious play in order to enable managers to approach and develop their activities differently ^4, 5^.

Lego® Serious Play® evolved over the ensuing years into a consulting technique utilized by businesses other than the LEGO Group, including non-profit organizations and groups of non-governmental organizations, as well as in governmental contexts.

Lego® Serious Play® is a method aimed at developing thinking, communication and solving complex business management problems through the use of Lego construction play. The aim is to foster creative thinking through team building activities based on the use of Lego bricks to create metaphors of one's organizational identity and experiences. Participants work through imaginary scenarios using Lego bricks, which is why this type of activity is referred to as "serious play".

The approach is based on the idea that learning that involves the use of mental and manual activities produces a deeper and more meaningful understanding of the world and its possibilities, arguing that participants develop the ability to communicate more effectively, to call on their imagination more easily and to approach their work with greater confidence, commitment and intuition.

The basic theory of Lego® Serious Play® is grounded in three fundamental theoretical areas: play, constructivism and constructionism, and imagination skills. This means that it is an activity that is constrained by time and location, organized according to a set of rules, conventions, or agreements between participants, not imposed by adults, and carried out by incorporating aspects of fantasy and creative imagination. Seymour Papert's theories ^6^, constructivism and constructionism, were inspired by Jean Piaget, who believed that learning is most effective when people are actively involved in creating something outside of themselves, such as a sandcastle, an automobile, a computer program, or a book. According to Kearney’s studies ^7^, imagination implies not one but at least three meanings: to describe something, to build something, and to challenge something. The creators of Lego® Serious Play® saw imagination as the singular ability of humans to “form images” or “imagine” something.

In accordance with the methodology of Lego® Serious Play®, the source of original strategies in companies is the interaction between these three types of imagination that make up strategic imagination. Lego® Serious Play® is a workshop where participants use specifically chosen Lego elements to create their own models in answer to the facilitator's questions. These models provide a framework for conversation in groups, knowledge sharing, issue solving, and decision-making ^2, 3^. This methodology is now widely employed in many sectors, even though it was initially intended to boost innovation and corporate success by facilitating meetings, communication, and problem-solving procedures in business settings ^2^.

The use of bricks for the creation of metaphors makes it easier to understand and solve complex issues. Focusing on bricks, not people, facilitates discussion and negotiation while reducing the burden of personal conflict.

In medicine and healthcare Lego® Serious Play® has been used to allow pharmacy students to learn pharmacology ^8^ *,* to train medical students in the practice of effective communication ^9^, to instruct them in patient-centered interviewing skills ^10^ and to improve reflective practice in medical education ^11^.

### The idea: Building a Lego model as a scenario to be debriefed by in training debriefers

After reading and studying the applications and implications of Lego® Serious Play® we had the idea to adopt the principles behind this method and we created a different application for it, by using the construction of a Lego brick model (a plane, a helicopter, a car, etc.) as a simulation scenario. 

Our idea was to imagine that the construction of a simple model with Lego bricks could in itself represent a scenario to be eventually commented on later in the debriefing. In practice, during debriefing training courses, the Lego session is used to let the participants practice different debriefing techniques.

We hypothesized the following advantages of doing a scenario with Lego bricks instead of with a manikin in a simulation room:

- The topic is not medical but neutral (building a model)
- The discussion is exclusively about behavioral aspects such as: Did someone take the leadership? Who decided which model to build and how to build it? Was the workload adequately distributed? Were periodic checks performed?
- Differences between participants are mitigated as everyone has an equal opportunity to do the job regardless of their real-life profession.
- It might happen that those who are used to taking leadership in their professional life because they are in a top position, are inexperienced in building the bricks so they must play the role of team participant rather than leader.
- And, vice versa, it might happen that those who in their professional life usually play the role of teammate, might take the leadership of the team because they are the most experienced in the field of Lego construction.
- Building a model has less emotional relevance than experiencing a high-fidelity scenario with a patient, even if it is simulated.
- For the novice debriefer, facilitating a discussion about building a Lego model is easier, less emotionally involving and more fun. This allows him to better focus on the technical aspects of the debriefing such as how to ask questions.
- Everyone focuses exclusively on the team’s behavior rather than on arguing about technical aspects or discussing the right application of clinical guidelines.

One of the major problems in training novice debriefers to debrief a high-fidelity simulation session is how to train them to focus on dialogue and communication techniques rather than engaging in an unstructured discussion of technical or behavioral skills ^12^. They are usually influenced by their professional background and inclined to immediately focus on the clinical events that occurred during the scenario rather than on the appropriate debriefing methodologies and communication strategies to be used ^13^. In the first learning phases, the novice should mainly practice and focus on the sequence of the debriefing phases and the type of questions to ask the scenario participants and on their non-verbal and paraverbal attitude. Dealing with technical, clinical, and behavioral aspects at the same time requires skills and training that one usually does not have when starting out as a facilitator. It is often easier to give feedback on technical skills than on behavioral ones.

The novice debriefer usually needs to practice how to ask open-ended questions, to master the dialectical approach to participants, and to practice investigating the behavior of scenario participants in a Socratic way, rather than worrying and getting too involved in clinical events or technicalities. Therefore, we utilized Lego bricks to build straightforward, standardized scenarios that debriefers can use to practice facilitating dialogue during training without having to get into specifics about clinical performance.

In reality, the scenario in this instance involves building a model out of Legos. Because this is a completely non-clinical and neutral topic, the trainee debriefer can focus solely on the debriefing technique (e.g., how to ask questions and how to investigate the participants' behavior).

Moreover, in this way, the debriefer in training discovers for himself how debriefing is a truly reproducible method that works even with scenarios that apparently have nothing to do with the usual clinical medical simulation.

In practice, during the train-the-trainer courses, four course participants act as participants in the scenario, and build the bricks model, and one other, in turn, acts as debriefing facilitator, supervised by an experienced instructor. Participants are asked to build a model using Lego® bricks according to simple and basic rules defined by the workshop leader.

The construction of the model with the bricks is itself a scenario, comparable to a high-fidelity simulation scenario, containing the same behavioral features. In fact, when a model must be built using the bricks, one must decide, even if not explicitly requested, which model to build, which pieces to choose according to the instruction manual, and one has to share with one's team colleagues the tasks, for example, how to build and how to choose the pieces. In addition, during the construction of the model, someone will most probably assume some form of leadership and some kind of more or less close-knit team will probably be formed.

The trainer-in-training may practice the debriefing by focusing on the relationships that develop between the participants during the construction of the model, such as:

- who decides which model must be built.
- what are the criteria for choosing the model to be built (random, competence-based, etc.)?
- whether the decision is shared with the group.
- if the tasks for the construction of the model are distributed and with which criteria.
- whether there is clear leadership or whether it is alternated or shared.
- whether basic continuous reassessment principles are applied, e.g., periodic re-evaluation of work done, closed communication, anticipation, and planning, etc.
- if everyone actively contributes to building.
- whether there is a change of pace after a certain time: acquisition or sharing of leadership or new leadership, more functional redistribution of roles, re-evaluation of planning, monitoring of time passing, etc.

The debriefer may also observe how the professional roles the participants have in real life may influence their behavior during the construction of the model. For example, it may happen that leadership is taken by those who are used to exercising it or, on the contrary, by those who show specific skills or who are more comfortable with building bricks. The learner-debriefer will facilitate the debriefing process supervised by an experienced tutor who, at the end of the session, will debrief the debriefer in training.

## Methods

### Ethical statement

In our region, simulation centers do not have access to a formal ethical approval process. However, even though we did not have the ability to apply to an ethics committee for this work we have carried it out according to the Declaration of Helsinki. Our simulation center adheres and follows the Healthcare Simulationist Code of Ethics supported by the Society for Simulation in Healthcare (Healthcare Simulationist Code of Ethics, 2018). Our study was eligible for an exemption, in accordance with US Federal Human Subject Regulations- Protection of Human Subjects, due to the nature of the study itself, as no patients were involved, the subjects participating were volunteers, researchers ensured that those taking part in the research would not be caused distress, all participants’ personal and other data were completely anonymized, and all the investigators had no conflict of interest and were not involved in any of the participants’ university teaching programs

### In practice

***One team play*.** Materials needed: a Lego 3-in-1 box such as Lego Creator® model 31071 (for children aged 6 to 12), which allows three models of increasing complexity to be built in 32, 39 or 53 steps.

Participants: four participants and the debriefer in training.

The four participants are invited to sit around a table and the debriefer explains the rules of the game: they must build the model as quickly as possible and in any case within a set time (usually 10 minutes).

It is not specified that the box of bricks contains three different models to be built, each of different difficulty and requiring a different number of bricks to complete the model. This gives the debriefer the opportunity to assess the initial decision-making processes: Does the team have a briefing before starting or is the choice of model built randomly? Does the team realize that there are three models or does the rush to start prevent a choice (as is often the case)? If the team is not able to complete the model in time and has chosen (perhaps by chance) the more difficult model, the debriefer will have the opportunity to point out that the lack of an initial briefing may have contributed to the failure to achieve the objective ("If we had chosen an easier model, we would have completed it in time!").

If you want two trainee-debriefers to practice at the same time, you can have two teams playing at the same time, each observed and then facilitated by a trainee-debriefer. The supervisor will supervise both exercises and give feedback to both debriefers in turn.

***Two teams playing without rules*.** Materials needed: two identical Lego 3-in-1 boxes such as Lego Creator® model 31071 (for children aged 6 to 12), which allows three models of increasing complexity to be built in 32, 39 or 53 steps.

Two teams of four participants each are arranged to sit around a table and the debriefer explains the rules of the game: they must build the model in the shortest time possible and in any case within a set time (usually 10 minutes). The team that finishes first wins. If time runs out before both teams have completed the model, the team with the fewest pieces left to assemble wins.

It is not specified that the box of bricks contains three different models to be built, of different difficulty and requiring different numbers of bricks to complete the model. This gives the debriefer the opportunity to assess the initial decision-making processes: does the team give a briefing before starting or is the choice of model to build random? Does the team realize that there are three models or does the rush to start preventing an initial assessment? If a team cannot complete the model in time and has chosen (perhaps by chance) the most difficult model, the debriefer will have the opportunity to point out that the lack of an initial briefing may have contributed to the failure to achieve the objective ("If we had chosen an easier model, we would have completed it in time and we would have won!").

***Two teams playing with rules*.** Materials needed: two identical Lego 3-in-1 boxes such as Lego Creator® model 31071 (for children aged 6 to 12), which allows three models of increasing complexity to be built in 32, 39 or 53 steps.

Two teams of four participants each are arranged to sit around a table and the debriefer explains the rules of the game: they must build the model in the shortest possible time and in any case within a set time (usually 10 minutes). Participants are informed that there are three models to build and that they can freely decide which model to build. The team that finishes first wins, but the team that decides to build the most complex model, which therefore requires more bricks to complete, receives a bonus of three minutes. If time runs out before both teams have completed the model, the team that has chosen the more complex model wins. In this way, the debriefer facilitates an initial debriefing, at least to decide which model to build: the team will have to make the decision whether it is more convenient to decide to build the easier model or to get a bonus. 

***One practical example*.** We summarize here the content of a real debriefing. Participants were all anesthesiologists working in the same hospital: Marta, Fiorella, Stefano and Catherine. Participants in this scenario had 10 minutes to complete the construction of a model made with Lego bricks. They were given a box containing the pieces needed to build the model and instructions on how to assemble it. They were free to choose which model to build among the three contained in the assembly instructions. They were not given any rules.

 After having received the box containing the Lego bricks, Marta immediately took the initiative and proposed to the group the model to build, and the others accepted without discussing the proposal. Stefano started reading the instructions. In the first 5–7 minutes the group did not seem particularly organized. Marta sometimes indicated the time already spent on the task. At a certain point, the group found some difficulties in assembling the model and everyone participated in solving the problem, making a checklist of the work done and stopping to take stock of the situation. The group did not manage to complete the model within the set time limit.

Nothing but positive emotions came out of the de-roleing and everyone expressed great satisfaction with the way things had worked. This would suggest that there were no critical issues.

However, the debriefer observed some criticalities during the scenario, including a) Marta took control of the situation by choosing the model to build, but her choice was not shared, and it was not clear on what it was based; b) there was no distribution of tasks, which were taken on autonomously and in a partial and confused way; c) the construction of the model was not completed.

During the analytical phase, Marta, who apparently acted as the leader, reported that as it was a question of building a model, she underestimated the work that needed to be done and that it would have been better to have a briefing before starting to build the model together. She also acknowledged that the same thing could happen in her situation: when dealing with a seemingly easy task there is a risk of underestimating its importance and neglecting the rules of good clinical practice. All participants agreed and said that on a future occasion they would have a briefing before starting to build the model to better organize the task, to prevent confusion, to define a clear distribution of work and cited the “distribute the workload” and the “continuous reassessment” (CRM) principles. For example, all the participants agreed that if they had done an initial briefing, they could have divided the tasks differently, for example one of them could have had the task of looking for the pieces, the other of cataloguing them, another could have read the instructions, yet another could have assembled the model, and this would have been quicker.

In addition, all the participants at the end of the analytical phase, recognized that if they had organized themselves before starting, they would also have discovered that there were three models having different difficulties of construction, and therefore they would have had the chance to choose differently and, most likely, to finish on time. One of the participants also recognized that this often happens to him during an emergency, when he goes straight to the patient and does something for him without thinking about it and without consulting his colleagues in the team first. In the application phase, the participants reported that they had learned the following points: the importance of establishing roles when doing teamwork; to stop for a while when there are moments that are a bit critical in order to double check, re-evaluate and resume work accordingly.

## Conclusions

The added value of learning how to facilitate using a "neutral" and "fun" scenario is that the trainer-in-training can apply the various debriefing methodologies, focusing exclusively on method and dialogue, without getting involved or having to concentrate on the technical aspects. In addition, building a model with Lego bricks is a perfect metaphor of the CRM principles.

We think that this method can be easily applied by any simulation centre. Apart from being used to facilitate the learning of novice debriefers, it may also be used as a surrogate for the scenario when you want the participants of a simulation course to exclusively improve the practice of non-technical skills and of the CRM principles as reported above in our practical example.

Based on our practical experience, it would be interesting to develop research aimed at demonstrating whether the repeated practice of a scenario with Lego bricks can facilitate and accelerate the learning of some basic principles of CRM, as eventually confirmed by subsequent scenarios and debriefings on simulated clinical cases.

## Consent

Written informed consent for publication of the participants’ details was obtained from the participants.

## Data Availability

No data are associated with this article.
