# Supplementary material for: Lego® bricks assisted training of the novice debriefers
Source: MedEdPublish (2016). 2023 Mar 20;13:16. [Version 1] doi: 10.12688/mep.19314.1 (PMC10076905; doi:10.12688/mep.19314.1)
Supplement: Peer review history

# Peer review: Reviewer response for version 1

**DOI:** 10.21956/mep.20695.r33033

**Published:** 2023-04-05

**Authors:** Carla Sa-Couto

**Affiliations:** 1 CINTESIS@RISE; Community Medicine, Information and Decision Sciences Department (MEDCIDS), Faculty of Medicine of University of Porto (FMUP), University of Porto, Porto, Portugal

Thank you for submitting this interesting paper, presenting an innovative approach that can be used in faculty development, namely in training novice debriefers. As a simulation facilitator leading train-the-trainer programmes, I greatly appreciated the clear and detailed description of the activity and examples of how to implement it. The paper is overall well-structured and is presented in a clear and accessible language.

 I do have some suggestions that I believe would be beneficial to the paper. I leave this to authors’ consideration.

- Although the paper focus on the use of serious play to train novice debriefers, this is not reflected in the “One practical example” section. This section describes the activity and the debriefing but does not address how this activity was used to train the novice debriefer. Considering the aim of the paper, it would be useful for the reader the description of the “debriefing-of-the-debriefing”, namely the learning objectives for the debriefer in training (e.g. followed a structured debriefing; psychological safety guaranteed; use of specific questioning technics, etc), and how was it conducted (informally, using an observational tool, using an objective assessment tool, etc).
- The “In practice” section has a considerable amount of redundancy. I suggest the authors to reorganize this section. A possible rearrangement is suggested below.
- In the description of the variations of the game, there is a contradiction in the text. Although the subsection title mentions “without rules”, the 2 ^nd^ paragraph mentions “debriefer explains the rules.” In my understanding there are rules in all variations of the game, but in same cases are given “additional instructions”. 
- Considering that the authors included a “Methods” heading, I would advise to also include a “Results” heading, before “One practical example”. Alternatively, I suggest to remove the “Methods” heading.
    
- There are some minor corrections needed (underlined):
  - Subsection “teams plays with rules” – The sentence “In this way, the debriefer facilitates an initial debriefing,…” should be “In this way, the debriefer facilitates an initial briefing,…”
  - Subsection “One practical example” – The sentence “We summarize here the content of a real debriefing.” Should be “We describe here the content of a real activity followed by debriefing.”
  - Subsection “One practical example” – The sentence “They were not given any rules” should be “ No additional instructions were given”.
  - Subsection “One practical example” – The sentence “She also acknowledged that the same thing could happen in her situation” should be “She also acknowledged that the same thing could happen in her clinical environment"

------

**Suggestion of reorganisation of the “In practice” section**

**In practice**

Materials needed (per team): a Lego 3-in-1 box such as Lego Creator® model 31071 (for children aged 6 to 12), which allows three models of increasing complexity to be built in 32, 39 or 53 steps.

Participants: four participants and the debriefer in training. This setting can be duplicated to two teams (further information below).

Setting: Teams of four participants are invited to sit around a table and the correspondent debriefer (one per team) explains the rules of the game: they must build the model as quickly as possible and in any case within a set time (usually 10 minutes). In case of multiple teams an additional rule is included: that the team that finishes first wins. If time runs out before both teams have completed the model, the team with the fewest pieces left to assemble wins.

 Each team will be observed and then facilitated by a trainee-debriefer. The supervisor will supervise the exercises and give feedback to the debriefers in turn.

 Variations

*Single or multiple teams playing without additional instructions*: It is not specified that the box of bricks contains three different models to be built, each of different difficulty and requiring a different number of bricks to complete the model. This gives the debriefer the opportunity to assess the initial decision-making processes: Does the team have a briefing before starting or is the choice of model built randomly? Does the team realize that there are three models or does the rush to start prevent a choice (as is often the case)? If the team is not able to complete the model in time and has chosen (perhaps by chance) the more difficult model, the debriefer will have the opportunity to point out that the lack of an initial briefing may have contributed to the failure to achieve the objective ("If we had chosen an easier model, we would have completed it in time!").

*Multiple teams playing with additional instructions: *Teams of four participants each are arranged to sit around a table and the debriefer explains the rules of the game: they must build the model in the shortest possible time and in any case within a set time (usually 10 minutes). Additionally, participants are informed that there are three models to build and that they can freely decide which model to build. The team that finishes first wins, but the team that decides to build the most complex model, which therefore requires more bricks to complete, receives a bonus of three minutes. If time runs out before both teams have completed the model, the team that has chosen the more complex model wins. In this way, the debriefer facilitates an initial briefing, at least to decide which model to build: the team will have to make the decision whether it is more convenient to decide to build the easier model or to get a bonus.

Is the rationale for developing the new method (or application) clearly explained?

Yes

Is the description of the method technically sound?

Yes

Are the conclusions about the method and its performance adequately supported by the findings presented in the article?

Yes

If any results are presented, are all the source data underlying the results available to ensure full reproducibility?

Not applicable

Are sufficient details provided to allow replication of the method development and its use by others?

Partly

Reviewer Expertise:

Simulation Educator and Researcher

I confirm that I have read this submission and believe that I have an appropriate level of expertise to confirm that it is of an acceptable scientific standard.

# Peer review: Reviewer response for version 1

**DOI:** 10.21956/mep.20695.r33036

**Published:** 2023-03-30

**Authors:** Fabrizio Consorti

**Affiliations:** 1 Faculty of Medicine and Dentistry, University Sapienza of Rome, Roma, Italy

The article describes the theoretical tenets and the method to use the Lego bricks in the training of debriefers of a simulation center.

 The topic is really interesting and the flow of the arguments is smooth. The method is clearly described and a worked example helps the reader to understand it better.

 I have only two suggestions. First, reshaping the Introduction. The problem (paragraph starting with "The trainer-in-training may practice the debriefing by focusing on the relationships..." and following 8 points) should come before, and the hypothesized solution ("We hypothesized the following advantages of doing a scenario...") after you declared that focusing on the relationship is a problem for the novice debriefer.

 Second, the description of the three modalities can be shortened without repeating the same sentences (e.g. materials and the context are the same as before ... In this modality two teams ...)

Is the rationale for developing the new method (or application) clearly explained?

Yes

Is the description of the method technically sound?

Yes

Are the conclusions about the method and its performance adequately supported by the findings presented in the article?

Yes

If any results are presented, are all the source data underlying the results available to ensure full reproducibility?

Not applicable

Are sufficient details provided to allow replication of the method development and its use by others?

Yes

Reviewer Expertise:

Medical education

I confirm that I have read this submission and believe that I have an appropriate level of expertise to confirm that it is of an acceptable scientific standard, however I have significant reservations, as outlined above.

# Peer review: Reviewer response for version 1

**DOI:** 10.21956/mep.20695.r33034

**Published:** 2023-03-27

**Authors:** Marc Lazarovici

**Affiliations:** 1 Institute for Emergency Medicine and Management in Medicine (INM), University Hospital, Ludwig Maximilians University, Munich, Germany

This description of using Lego bricks to enhance the capabilities of novice debriefers is quite interesting and innovative. I believe both the rationale and the method are very clearly described, along with a good example of practical usage.

 There are some details that might improve on the quality of the article.

 First, the paragraph "The approach is based on the idea that learning that involves the use of mental and manual activities produces a deeper and more meaningful understanding of the world and its possibilities, arguing that participants develop the ability to communicate more effectively, to call on their imagination more easily and to approach their work with greater confidence, commitment and intuition." is lacking a proper source - adding one will help the reader understand the basis of this affirmation.

 In the next paragraph I think replacing "adults" by "others" is more applicable to the context of the article, as we are not talking about children.

 Towards the end of the article, there are two almost identical paragraphs - both starting with "It is not specified that the box of bricks contains three different models to be built,..." Probably the first one would suffice, while the second could be replace by directing the reader to the first description.

Is the rationale for developing the new method (or application) clearly explained?

Yes

Is the description of the method technically sound?

Yes

Are the conclusions about the method and its performance adequately supported by the findings presented in the article?

Yes

If any results are presented, are all the source data underlying the results available to ensure full reproducibility?

No source data required

Are sufficient details provided to allow replication of the method development and its use by others?

Yes

Reviewer Expertise:

Simulation training and education

I confirm that I have read this submission and believe that I have an appropriate level of expertise to confirm that it is of an acceptable scientific standard.
